# Prospective Screening of Family Members with Moyamoya Disease Patients

**DOI:** 10.1371/journal.pone.0088765

**Published:** 2014-02-19

**Authors:** Cong Han, Hui Feng, Yi-Qin Han, Wei-Wei Liu, Zheng-Shan Zhang, Wei-Zhong Yang, Lian Duan

**Affiliations:** 1 Department of Neurosurgery, 307 Hospital, PLA, Beijing, China; 2 Department of Ultrasound, 302 Hospital, PLA, Beijing, China; 3 Consulting centre of biomedical statistics, academy of military medical sciences, PLA, Beijing, China; Anhui Medical University, China

## Abstract

**Background:**

Genetic factors play an important role in the pathogenesis of moyamoya disease (MMD). Previous studies concentrated on familial MMD patients. In this study, we focused on family members of sporadic MMD patients, and aimed to gain a clearer understanding of the role that genetic factors play in MMD.

**Methods:**

The immediate family members of MMD patients were initially screened by transcranial Doppler sonography (TCD) and positive cases were verified by magnetic resonance angiography (MRA).

**Results:**

From July 2011 to March 2013, there were 527 MMD patients managed in our hospital, including 38 familial MMD cases. In this study, 285 immediate family members of 245 sporadic MMD patients were screened. Another 41 cases of familial MMD cases were identified, which included 21 family members and 20 corresponding sporadic MMD patients who had family members confirmed positive with MMD. As a result, the proportion of familial MMD patients increased from 7% (38/527) to 15% (79/527) in this period. For the main segments of the circle of Willis, Kappa values between TCD and MRA for the anterior cerebral arteries, middle cerebral arteries and posterior cerebral arteries were 0.91, 0.72, and 0.47, respectively. Familial cases confirmed by our screening showed a significantly higher percentage of asymptomatic patients (57%) compared with 9% from the control group who had a clear family history before.

**Conclusions:**

Familial MMD patients may account for a higher percentage among all cases than previously thought. Some family members of MMD patients may also have MMD, but not have any obvious symptoms. Routine screening should be implemented for all family members of MMD patients to improve the detection rate for this part of the patient base. TCD has a high diagnostic agreement with MRA for MMD. TCD may be the preferred choice for screening because it is inexpensive and safe.

## Introduction

Moyamoya disease (MMD) is a chronic cerebrovascular disorder, characterized by progressive stenosis and/or occlusion of the intracranial internal carotid artery and its proximal branches, with development of a basal collateral network. Previous studies have suggested that genetic factors play an important role in the pathogenesis of MMD [1]. Approximately 6–12% of MMD patients have a family history [2]–[3]. However, no screening of family members for sporadic MMD cases has been reported. Therefore, in this study, we screened the family members of MMD patients who do not have an established family history. Our result may help further clarify the role that genetic factors play in MMD. In addition, we also aimed to evaluate the effectiveness of transcranial Doppler sonography (TCD) in MMD screening.

## Subjects and Methods

### Patient Selection

From July 2011 to March 2013, there were 527 MMD patients managed in our hospital, including 38 familial MMD cases. In this study, 38 confirmed familial cases (from 17 families) were chosen as control group. For the other sporadic MMD patients, 4 family members of 2 MMD patients were excluded for absent temporal bone windows to screen with TCD, and other family members of 242 patients refused to participate for a variety of reasons. Therefore, 285 immediate family members (first degree relative only) of 245 sporadic MMD patients were enrolled. None of the family members had a previous diagnosis history of MMD or any other cerebrovascular disease. Subjects who had other basic diseases, such as arteriosclerosis, autoimmune disease, meningitis, Down syndrome, and so on, were excluded from the study, in accordance with the guidelines for diagnosis and treatment of MMD in 2012 [4].

### Examination

All family members were initially screened with TCD. A transcranial Doppler instrument (DWL Doppler-Box, Germany) was used to examine the subjects according to a previously published standard protocol [5]–[6]. All of the screening was performed by an experienced sonographer in TCD. When a suspected case occurred, magnetic resonance angiography (MRA) was performed to validate the diagnosis of MMD by the 3-dimensional time-of-flight method with a 1.5 T superconducting magnet (Signa, GE Medical Systems). The diagnostic criteria of MRA was based on the guidelines recommended by the Research Committee on the Pathology and Treatment of Spontaneous Occlusion of the Circle of Willis in 2012 [4].

### Statistical Analyses

Data are shown using frequencies and percentages for categorical variables, and means and standard deviations for continuous variables. The consistency between the results of TCD and MRA was calculated by the chi-square test and Kappa testing. Comparisons between family members (screened group) and the control group was evaluated using the chi-square test and t test. The factors analyzed included sex, age, family relationship, and clinical symptoms. Statistical analyses were performed with SAS 9.2 (SAS Institute Inc, Cary, NC). Statistical significance was set at P<0.05.

### Ethics Statement

This study was approved by the local ethical committee (the Ethics Committees of 307 Hospital, PLA), and written informed consent forms were obtained directly from patients and the immediate family members.

## Results

**Figure 1:**
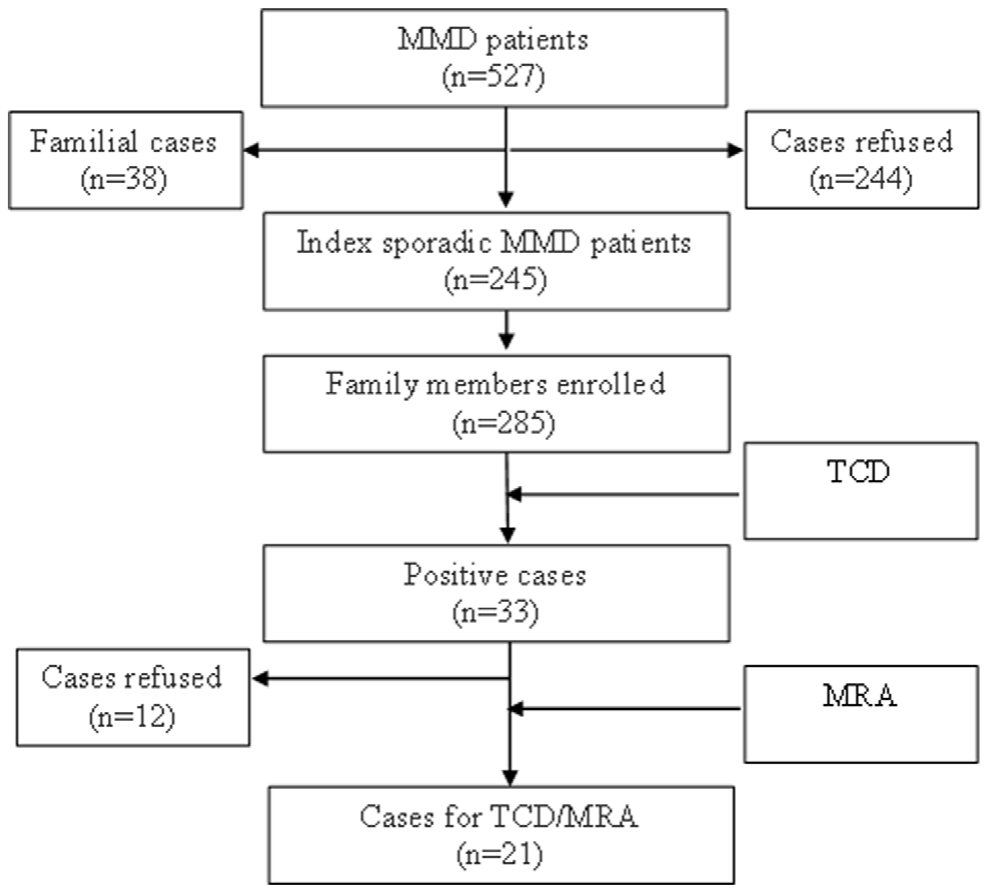
Diagram of Family members with MMD included in this study.

Abnormal results were found in 33 subjects from 285 immediate family members by TCD screening, with an 11.6% detection rate. 21 of the 33 subjects underwent a MRA to make a definite diagnosis. The other 12 subjects refused to have further examinations (Figure 1). In the 21 cases who were examined further, the accordant rate between TCD and MRA was 100%. 11 cases were diagnosed with typical bilateral MMD, and the other 10 cases were diagnosed with unilateral MMD. We also evaluated the parameters of the 3 main intracranial arterial segments - the anterior cerebral arteries (ACA), middle cerebral arteries (MCA) and posterior cerebral arteries (PCA) in the circle of Willis - in all 21 cases (32 sides). Table 1 shows contingency tables for the categorical variables used for the assessment of stenosis. The results of TCD and MRA were highly consistent. Kappa values for the comparison of TCD and MRA were 0.92 for the ACA, 0.72 for the MCA, and 0.47 for the PCA. Such high Kappa values in the ACA and MCA indicated substantial agreement between the two methods, and TCD findings matched MRA findings in 94 of 96 arterial segments (94.8%). The sensitivity and specificity of TCD were 96.3% and 92.9% respectively, compared with MRA.

**Table 1:** Main results of TCD compared to MRA.

TCD		ACA		MCA		PCA	
	MRA	P	N	P	N	P	N
**P**		24	0	27	2	1	1
**N**		1	7	0	3	1	29
**K value**		0.92		0.72		0.47	

(ACA = anterior cerebral arteries, MCA = middle cerebral arteries, PCA = posterior cerebral arteries, TCD = transcranial Doppler sonography, MRA = magnetic resonance angiography, P = positive, N = negative, K = Kappa).

Prior to our study, the proportion of familial MMD in our hospital was 7.2% (38/527) from July 2011 to March 2013. In this screening, we found that 21 family members and 20 corresponding index MMD patients (the index patients are the confirmed MMD cases whose family members were screened in this study) also had family heredity of MMD. Therefore, the proportion of familial MMD increased to 15.0% (79/527). In all 21 positive cases, the male/female ratio was 11/10, and the age ranged from 7 to 55 years old, with a mean age of 35 years old. The genetic relationships between the screened subjects and index patients were 6 father-offspring pairs, 7 mother-offspring pairs and 8 sibling pairs. The clinical symptoms of the positive screened cases included TIA (transient ischemic attack, 5 cases), cerebral infarction (4 cases), and the other 12 cases were asymptomatic. There were no significant differences in age, sex, or genetic relationship between the screened groups and the control group. However, a significantly higher percentage (57%, 12/21) of asymptomatic cases was observed in the screened group compared with the confirmed familial MMD group (9%, P<0.001). In addition, the proportion of those presenting with severe symptoms, including infarction and hemorrhage, was higher in the confirmed familial MMD group than in the screened group (Tables 2).

**Table 2:** Characteristics of family members screened for moyamoya disease.

Characteristics	Cases		Significance (P value)
	family members screened	control	
**Confirmed cases**	21	38	
**Male/Female**	11∶10	23∶15	0.5444
**Age (years)**	35±12	32±16	0.4086
**Relationship**			
*father–offspring*	6	9	0.3232
*mother–offspring*	7	3	
*siblings*	8	9	
**Clinical symptoms**			
*asymptomatic*	12	2	0.0001
*headache*	0	7	
*TIA*	5	12	
*infarction*	4	13	
*hemorrhage*	0	4	

(TIA = Transient Ischemic Attack).

## Discussion

MMD used to be considered a rare cerebrovascular disorder and the number of cases reported is scarce. With the development of noninvasive diagnostic tools such as MRA and growing awareness by clinicians, the detection rate of MMD has dramatically improved. A nationwide epidemiological survey in 2003 showed that the prevalence and incidence of MMD in Japan was 6.03 and 0.54 per 100 000, respectively [2]. An even higher prevalence has been shown by some regional surveys at 9.1–10.5 per 100,000 [7]–[8].

The exact etiology and pathogenesis of MMD remain unknown, but various evidence has indicated that genetic factors play a major role [1], [3]. A total of 12.1% of patients have a family history of MMD according to a Japanese epidemiological survey [2], and 6% were reported in the USA [3]. However, all of the previous data for familial occurrence were based upon well-defined familial patient groups, and there has been no study focusing on family screening of sporadic cases. In our study, we prospectively examined 285 immediate family members of 245 MMD patients using TCD. We found that 7.4% (21/285) of cases were confirmed as positive for MMD. Consequently, the proportion of familial MMD in our hospital increased from 7.2% (38/527) to 15.0% (79/527). Because the index patients for this study only comprised 245 out of the 527 MMD cases in this period, the proportion may be higher than 15%. Therefore, the proportion of familial MMD may be higher than previously thought. Further prospective studies may be necessary to finalize management guidelines for family screening of sporadic MMD cases.

We also compared the clinical features between screened familial MMD cases in this study and confirmed familial MMD cases with a clear MMD family history in the past. We found that the majority of screened cases were asymptomatic (12/21) compared with the control group (2/38). This asymptomatic phenomenon might be the reason why some familial MMD patients are easily missed. Our findings also suggest that the incidence of asymptomatic MMD and the nature history of MMD need to be re-evaluated. Asymptomatic MMD patients have not previously received enough attention, they are often hard to detect and the nature history was unknown. However, increasing evidence suggests that disease progression for these patients is severer than previously considered. A multicenter questionnaire survey including 33 asymptomatic MMD patients showed that, in the conservatively treated group (28 patients), 2 patients died from intracranial bleeding and 4 patients experienced TIA during follow-up period of an average of 44 months [9]. Another multicenter survey included 34 non-surgically treated asymptomatic patients, 3 patients experienced TIA, 1 patient had ischemic stroke and 3 patients had intracranial bleeding at follow-up. The annual risk for all kinds of strokes was 3.2% during follow-up period [10]. These previous results show that asymptomatic MMD is not a silent disorder, and disease progression might occur silently or continue to be undetected until it presents with serious neurological deficits. Asymptomatic MMD patients should be carefully monitored for disease progression before a serious cerebrovascular event occurs. Therefore, we recommend that all immediate family members of MMD patients should be screened by neuroimaging, regardless of their family medical history. Such a routine vascular screening may lead to early surgical intervention and prevention of significant neurological complications. Because the financial cost of routine screening may be significant, TCD is a cost effective choice.

Digital subtraction angiography (DSA) is still the golden standard for the diagnosis of MMD at present, but MRA findings are also recognized as a diagnostic criteria [4], [11], [12]. In addition, TCD is another diagnostic tool, which is widely used to identify different intracranial vascular abnormalities. Many studies have reported the diagnostic value of TCD compared with MRA, computed tomographic angiography (CTA), and DSA [13]–[14], but a limited number of studies have investigated its application in MMD. Lee et al [5] detected 45 adult MMD patients with TCD, while all the patients were diagnosed with MRA or DSA. They showed that changes in TCD parameters can reflect the severity of MMD, and TCD results can be coordinated with MRA findings. In our study, the Kappa statistic showed high consistency between these two methods in the ACA and MCA. The sensitivity and specificity of TCD was 96.3% and 92.9%, respectively. Therefore, our study and previous studies show that TCD is an effective method for MMD screening.

However, TCD has some limitations. First, in MMD, lesions are limited to the circle of Willis, which has confluent flowing from the internal carotid arteries and the basilar artery. The hemodynamics in this area are significantly different anatomically from normal branching, such as the distal branches of the MCA or PCA. Especially in the ACA, the results are usually affected by the contralateral ACA through the anterior communicating artery. A great deal of experience of operators is required to identify it. Second, TCD examinations depend on the cranial “window” where the ultrasound beam can penetrate without being excessively dampened, but some cases do not have a corresponding “window”. Therefore, currently, TCD cannot replace DSA or MRA. However, in terms of screening a high-risk population, TCD shows a number of advantages over other available methods because it is non-invasive, non-ionizing, inexpensive, portable, and safe.

In conclusion, our study shows that a considerable portion of family members of sporadic MMD patients also have MMD. The proportion of familial MMD patients may be higher than previously thought and this issue requires re-evaluation. Because this part of MMD patients is asymptomatic or only presents with mild symptoms, sufficient attention and in-depth analysis have not been applied in the past. Routine vascular screening with necessary follow-ups for all immediate family members of MMD patients is highly recommended. These processes may lead to early detection and prevention of severe complications. TCD is considered a good choice as a screening method because it is cost effective, and is non-invasive with good acceptance, among other advantages. Because no reliable criteria for TCD parameters have been set for the diagnosis of MMD, all suspected cases detected by TCD currently require further examinations to confirm the diagnosis and severity.
